# Evaluation and remediation protocol of selected organochlorine pesticides and heavy metals in industrial wastewater using nanoparticles (NPs) in Nigeria

**DOI:** 10.1038/s41598-023-28761-3

**Published:** 2023-02-07

**Authors:** Jude Chidozie Nnaji, James Friday Amaku, Okoche Kelvin Amadi, Solomon Ireji Nwadinobi

**Affiliations:** Department of Chemistry, Michael Okpara University of Agriculture Umudike, P.M.B 7267, Umuahia, Abia Nigeria

**Keywords:** Environmental sciences, Chemistry, Materials science, Nanoscience and technology

## Abstract

Limited knowledge of the level of contaminants in industrial wastewater within the Nigerian states together with the global challenge of water supply have compelled our investigation into the analyses and removal of organochlorine pesticides (OCPs) and heavy metal contents in industrial wastewater. Wastewater samples were collected from 13 industries across five states in Nigeria. The OCPs content of the samples was extracted, cleaned up and analysed using gas chromatography-mass spectrometry. The results indicate that the mean concentrations of the OCPs in the effluent samples ranged from 1.76 ng L^−1^ (Dieldrin) to 0.89 ng L^−1^ (endrin). Cadmium (Cd), chromium (Cr) and lead (Pb) were evaluated in all the effluent water samples. The results show that the average concentrations of the heavy metal ions in the effluent samples ranged from 0.008 ± 0.003 mg L^−1^ (Cd) to 2.215 ± 0.841 mg L^−1^ (Pb). For the removal of the identified contaminants, biomagnetite nanoparticles (BioMag), magnetite nanoparticles (MagNPs), biomagnetite-CMC nanocomposite (BioMag-CMC) and magnetite-CMC nanocomposite (MagNPs-CMC) were synthesised and characterised using Braunauer–Emmett–Teller (BET), Fourier transform infrared (FTIR) spectroscopy, X-ray diffraction (XRD) and high resolution-transmission electron microscopy (HR-TEM). This study demonstrates the successful application of nanoparticles (NPs) and nanocomposites in the removal of OCPs and heavy metal ions in industrial effluents. The routine assessment and continuous removal become important to attain a state of clean and healthy aquatic ecosystem due to rapid industrial and technological advances.

## Introduction

Pollution caused by OCPs and heavy metals discharged into the waterbodies from food, tannery, personal care product, malting, textile, pesticide, brewery, mining, paint, cement, fertilizer and pharmaceutical industries is on the increase and has posed danger to the well-being of man and the environment^1,2^. Organochlorine pesticides (OCPs) are known to sustain their toxicity for long period in the environment^3^. Meanwhile, long-term exposure to OCPs and their metabolites have been reported to cause devastating health implications such as reproductive system dysfunction, neurological impairment, dysfunctional immune system, birth defect and cancer^4–6^. On the other hand, heavy metals have demonstrated the capacity to induce illnesses such as disorders of the nervous system, cancer, organ damage, and in extreme cases, death^7,8^. Hence, it is essential to eliminate these classes of water contaminants from wastewater before discharge. To achieve this, water treatment techniques such as solvent extraction, and ion-exchange processes^9^, chemical precipitation^10^, chemical oxidation or reduction^11^, membrane technology^12^, filtration^13^, electrochemical treatment^14^, adsorption^15–18^, foam separation^19^ and photocatalysis^20,21^ have been used for the remediation of contaminated water. Among the aforementioned techniques, adsorption is economical, user-friendly and effective for contaminants sequestration. Adsorbents such as molecular sieve^22^, rice husk^23^, granite^24^ Scots pine^25^, silica gel^26^, kaolinite clay^27^ and Al/SrTiO_3_^28^ amongst others have been used for the removal of these contaminants.

Nanomaterials, nanoparticles and nanocomposites have become a fast-growing and rapidly expanding area of scientific research due to their diverse applications in many areas of scientific and technical endeavour. Environmental concerns have also led to a growing interest in the green or biological synthesis of metal nanoparticles since the process reduces the use of chemical raw materials leading to lower disposal and incidence of chemicals in the environment. Nanoparticles are natural or engineered substances which have structural components whose sizes are less than 100 nm in three dimensions^29–31^. Nanoparticles are used in diverse fields which include medicine and drug delivery, environmental remediation, electronics and metallurgy^32,33^. Several works have reported successful nanoparticle biosynthesis with plant extracts^34–37^. Meanwhile, the application of nanometals in the wastewater treatment process has been extensively assessed^38–42^. A nanocomposite is a composite material made by combining two or more phases which contain different compositions or structures with at least one of the phases in the nanoscale range^43,44^. Nanocomposites enhance the macroscopic properties of the resultant products but the properties of nanocomposites are a function of the properties of the individual components. Bio-based nanocomposites are made with biodegradable or renewable materials like cellulose^45^. *Dalium guineense* is a woody plant of the rainforest zone of West Africa that may grow up to 10 to 20 m. Its common names include Black Velvet Tarimand in English, Icheku in Igbo, Awin in Yoruba and Tamarinier noir in French. The mature tree has a grey-coloured bark, dense green leaves and whitish flowers which bear the velvet black-coloured fruits which are seasonal and popular in West Africa and are a rich source of vitamins^46^. Cellulose is the most abundant natural polymer and numerous types of modified cellulose nanomaterials have been made using different methods^47–49^.

In this research, magnetite nanoparticles were synthesised chemically using iron (III) chloride hexahydrate. Similarly, the ethanolic and aqueous extracts of the stem bark of *D. guineense* were used for the biosynthesis of magnetite nanoparticles. The nanoparticles were incorporated in situ into carboxymethyl cellulose (CMC) to generate the nanocomposites. The bio-nanoparticles and nanocomposites were characterised and applied for the removal of identified metals (Cd, Cr and Pb) and OCPs (alpha(α)-BHC, beta(β)-BHC, gamma(γ)-BHC, delta(δ)-BHC, heptachlor, heptachlor epoxide, aldrin, gamma(γ)-chlordane, alpha(α)-chlordane, endosulfan I, endosulfan II, endosulfan sulfate, P,p'-DDE (dichloro-diphenyl chloroethane), dieldrin, endrin, endrin ketone, P,P′-DDD (dichlorodiphenyldichloroethane), P,P′-DDT (dichlorodiphenyltrichloroethane), endrin aldehyde and methoxychlor) from 13 different wastewaters.

## Materials and methods

### Magnetite nanoparticles (MNPs)

Magnetite nanoparticles were prepared by chemical precipitation as described by Khalil^50^; FeCl_3_·6H_2_O (19.46 g, 0.799 M) was completely dissolved in 150 mL distilled water to prepare aqueous solution A. Further, 6.584 g (0.792 M) of potassium iodide was dissolved in 50.0 mL of distilled water to prepare aqueous solution B. Solutions A and B were then mixed at room temperature, stirred and allowed to attain equilibrium for 1 h. The precipitate of iodine was filtered out and washed with distilled water. The washing was added to the filtrate and the whole volume was then hydrolysed using 25% ammonia solution which was added drop-wise with continuous stirring until complete precipitation of the black magnetite at a pH of 10. The suspension was allowed to settle, filtered, and washed with distilled water and the solid material was dried at 100 °C for 2 h.

### Collection and identification of *Dalium guineense* stem bark samples

**Figure 1:**
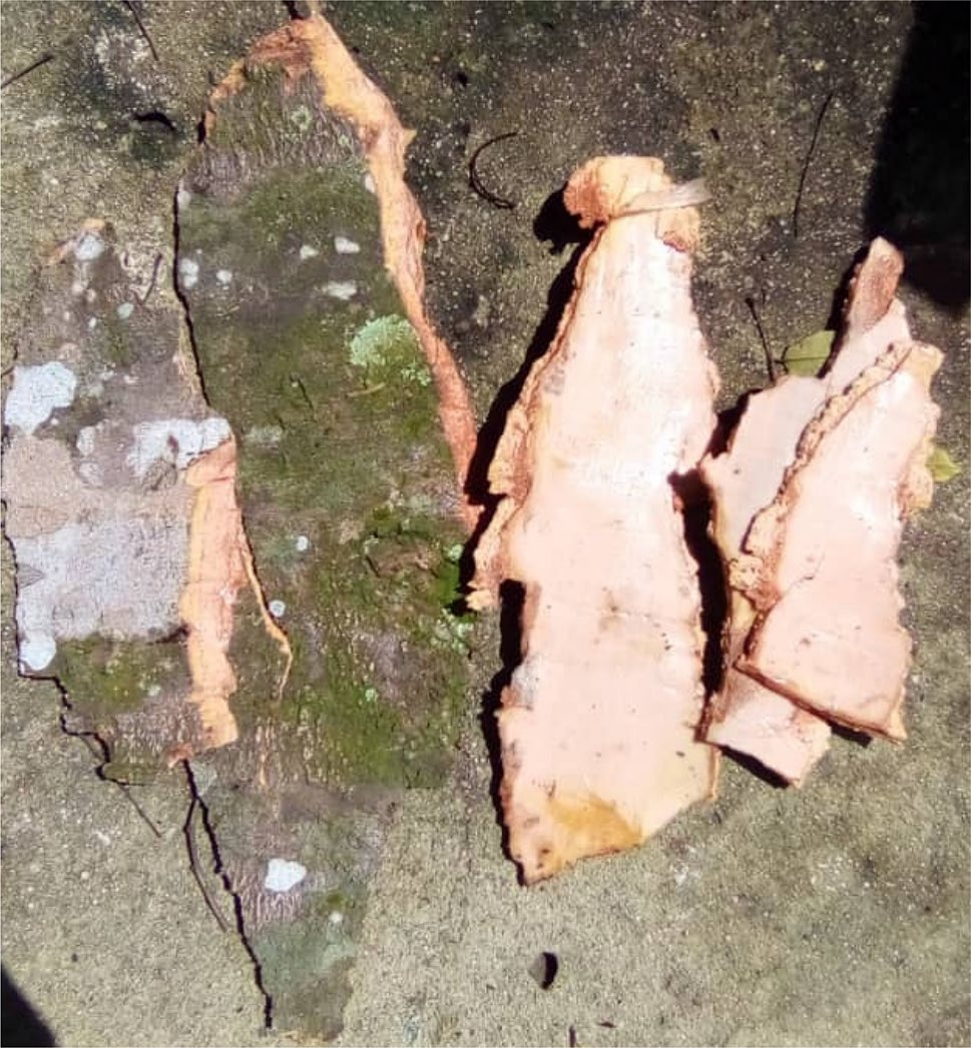
The stem bark of *Dalium guineense.*

The stem bark samples were collected within and around Michael Okpara University of Agriculture, Umudike, Abia State, Nigeria (see Fig. 1). They were tightly packed into plastic bags and transferred to the laboratory and identified at the Forestry Department of the University. The voucher specimens were deposited in the herbarium of the Plant Science and Biotechnology (PBS) Department of the same University and were declared not endangered. Samples were washed thoroughly 3 times with distilled water and were shade dried for 14 days. The dry samples (stem bark of *Dalium guineense*) were pulverized into powder with a wooden mortar and pestle. The ethanol extract was made with the modified cold maceration method of Azwanida^51^ by soaking 40 g of the powder in 200 mL of absolute ethanol for 20 h followed by filtration under vacuum through Whatman no 1 filter paper spread on a fitting Buchner funnel. The aqueous extract was made by heating 40 g of the powder in 200 mL of distilled-deionized water on a hot plate at 60 °C for 20 min, after which it was cooled and filtered through Whatman no. 1 filter paper. The extracts were used for nanoparticle biosynthesis.

### Biosynthesis of magnetite nanoparticles (Bio-Mag)

This was carried out with the modified method of Karnan^52^. FeCl_3_·6H_2_O and FeSO_4_·4H_2_O (1:2 molar ratios) were dissolved in 100 mL of double deionized water (DDW) in a 250 mL beaker and heated to 80 °C with mild stirring using a magnetic stirrer. The ethanol extract of the plant was added after 10 min resulting in a dark colour change. Aqueous NaOH (1 M, 10 mL) was also added after 10 min at the rate of 3 mL min^−1^ with continuous stirring to allow the magnetite to precipitate uniformly. The dark mixture changed to black suspended particles from the first addition of NaOH. The mixture was allowed to cool to room temperature and the residue (magnetite) was obtained by filtration through Whatman no 1 filter paper. The residue was washed 3 times each, with double distilled water and ethanol separately and dried at 80 °C for 2 h^52^.

### Chemical synthesis of magnetite-CMC nanocomposite (Mag-CMC)

Carboxymethyl cellulose (CMC, 1 g) was dissolved in 150 mL of 0.799 M solution of FeCl_3_·6H_2_O which was subsequently mixed with 50 mL of 0.792 M solution of KI with constant stirring. The mixture was allowed to equilibrate for 1 h and was filtered through Whatman no 1 filter paper and washed with distilled water. The filtrate was hydrolyzed with aq. NH_3_ to precipitate modified magnetite nanoparticles (MNPs) at pH 10. The precipitate was allowed to settle, the upper layer was decanted and the residue was filtered and washed with distilled water and dried at 60 °C for 5 h.

### Biosynthesis of biomagnetite-CMC nanocomposite (BioMag-CMC)

FeCl_3_·6H_2_O and FeSO_4_·4H_2_O (1:2 molar ratios) were dissolved in 100 mL of DDW in a 250 mL beaker and heated to 80 °C with mild stirring using a magnetic stirrer. Ethanol extract **(**20 mL) was added after 10 min with constant stirring followed by the addition of 1 g of CMC with constant stirring. After another 10 min, 20 mL of 1 M NaOH solution was added at the rate of 2 mL min^−1^. The precipitate was cooled to room temperature and dried in an oven at 80 °C for 2 h.

### Characterisation of nanoparticles

The synthesised nanomaterials were further characterised and confirmed. X-ray diffraction (XRD) measurements were performed using a multi-purpose X-ray diffractometer (D8-Advance from Bruker, USA) with LynxEye position sensitive detector operated in a continuous θ–θ scan in locked coupled mode with Cu-Kα (λKα_1_ = 1.5406 Å) radiation. Infrared spectra were obtained using a Fourier transform infrared spectrometer (FTIR, PerkinElmer Spectrum RX 1 spectrometer, PerkinElmer Inc., USA). High-resolution transmission electron microscopy (HRTEM) analysis was used to assess the surface structure and morphologies of the synthesised nanoparticles. Measurements with HRTEM (JEOL TEM 2100, JEOL Ltd, Japan) were carried out at an accelerating voltage of 100 kV. The specific surface area of the nanoparticles was acquired by making use of the Brunauer–Emmett–Teller (BET) nitrogen sorption–desorption method (Micromeritics Instruments Corp., USA). The pore volume and pore diameter were estimated by making use of the Barrett–Joyner–Halenda (BJH) theory.

### Sample collection

Wastewater samples were collected in triplicate at the sampling points (wastewater tanks or discharge pipes) of each of the 13 industries (Table 1) with 10 L acid-washed polyethylene gallons. An aliquot of each sample was measured into 1 L polyethylene bottle acidified with concentrated HNO_3_ (2 mL L^−1^) and reserved for metal analysis. Another set of aliquots was collected in pretreated 2.5 L amber reagent bottles, which were cleaned by making use of distilled water, acetone, and n-hexane. The amber reagent bottles were corked tightly, labelled appropriately and packed into ice chests before being transported to the lab for the relevant pesticide analysis. To prevent the degradation of the analyte, samples were stored at 4 °C prior to extraction.

**Table 1:** Wastewater types and location of Industries.

SN	Industry/wastewater	Condition of wastewater	State/city
1	PCP (PCUWA)	Untreated	Abia/Aba
2	Textile (TUWK)	Untreated	Kano/Kano
3	Soft drink (SUWK)	Untreated	Kano/Kano
4	Malting (MUWA)	Untreated	Abia/Aba
5	Pesticide (PTWK)	Treated	Kano/Kano
6	Tannery (TUKW)	Untreated	Kano/Kano
7	Brewery (BTWR)	Treated	Rivers/Port Harcourt
8	Paint (PUWA)	Untreated	Abia/Aba
9	Pharmaceutical (PTK)	Treated	Kano/Kano
10	Fertilizer (FTWR)	Treated	Rivers/Port Harcourt
11	Fibre Cement Board (FCTWE)	Treated	Enugu/Enugu
12	Coal Mine (CMTWM)	Treated	Gombe/Maiganga
13	Cement (CTWA)	Treated	Gombe/Ashaka

*PCP* Personal care product, effluent from PCP industry in Abia (PCUWA), effluent from textile industry in Kano (TUWK), effluent from soft drink industry in Kano (SUWK), effluent from malting industry in Abia (MUWA), effluent from pesticide industry in Kano(PTWK), effluent from tannery industry in Kano (TUKW), effluent from brewery industry in Port Harcourt (BTWR), effluent from paint industry in Abia (PUWA), effluent from pharmaceutical industry in kano (PTK), effluent from fertilizer industry in Port Harcourt (FTWR), effluent from fibre cement board industry in Enugu (FCTWE), effluent from coal mine industry in Gombe (CMTWM), effluent from cement industry in Gombe (CTWA).

### Preparation of stock solutions

The stock solution (1000 mg L^−1^) of each metal (Cd, Cr and Pb) was prepared with the appropriate metallic salts[Cd(NO_3_)_2_·4H_2_O, K_2_Cr_2_O_7_ and Pb(NO_3_)_2_]. These were used to prepare five standard solutions (0.25, 0.5, 5, 15 and 30 mg dm^−3^) of each metal by serial dilution. The standard solutions were aspirated into a flame atomic absorption spectrophotometer (AAS) (Varian SpectrAA 100) and the absorbances obtained were used in plotting calibration curves. Wavelengths for AAS analyses were 228.9 nm for Cd (R^2^ = 0.9997), 357.9 nm for Cr (R^2^ = 0.9989) and 283.2 nm for Pb (R^2^ = 0.9999). Detection limits for the metals were 0.001 mg L^−1^ for Cd, 0.1 for Cr and 0.01 mg L^−1^ for Pb^53^.

### Digestion of samples

Wastewater samples were digested according to Birtukan and Gebregziabher method^54^. A 50 mL filtered aliquot of water sample was pipetted into a digestion flask and digested with 3 mL concentrated HNO_3_ and 3 mL H_2_O_2_ at 70 °C for 1 h until a clear solution was obtained. The cooled clear solution was filtered through a Whatman no. 42 filter paper into a 50 mL volumetric flask which was made up to mark with distilled-deionized water. Blank digestion was also carried out in the same way with distilled-deionized water and the acids. The digested samples were analysed on a Varian SpectrAA 100 flame atomic absorption spectrophotometer^54^.

### Sample extraction

Prior to the extraction step, all glassware was cleaned and stored in the oven at 120 °C. The liquid–liquid extraction technique as provided by USEPA method 3510-C was used for analytes extraction^55^. Suspended dirt in the industrial wastewater was eliminated using the filtration technique. Thereafter, a 500 mL aliquot was extracted using 40 mL dichloromethane (DCM). The process was repeated three times and the extract fractions were combined and concentrated to 2 mL by making use of a rotary evaporator.

### Separation (clean‑up)

A silica gel (1000 mg/6 mL) column that is capped with anhydrous sodium sulphate (2 g, Na_2_SO_4_) conditioned with 6 mL dichloromethane was employed for the clean-up step. The concentrated extracts were loaded and eluted with a total volume of 50 mL of n-hexane:DCM:toluene in the ratio 2.5:1.5:1. The fractions of eluents collected were combined and concentrated to dryness using rotary evaporation. Thereafter, the extracts were re-dissolved in 2 mL n-hexane prior to GC–MS quantification.

### GC–MS analysis

An Agilent 6890N gas chromatograph equipped with an autosampler connected to an Agilent Mass Spectrophotometric Detector was used. 1 µL of the sample was injected into the pulsed spitless mode onto a 30 m × 0.25 mm ID DB 5MS coated fused silica column with a film thickness of 0.15 µL. Helium gas was used as a carrier gas and the column head pressure was maintained at 20 psi to give a constant of 1 mL min^−1^. Other operating conditions were pre-set. The injector and detector temperatures were set at 270 and 300 °C respectively. The oven temperature was set as follows: 70 °C held for 2 min, ramp at 25 °C min^−1^ to 180 °C, held for 1 min, and finally, ramp at 5 °C min^−1^ to 300 °C. All the quality control steps were followed.

### Determination of physicochemical parameters

The Hanna multi-parameter test meter (model, HI98194) was used to determine pH, temperature, electrical conductivity (EC) and total dissolved solids (TDS) after the probes were calibrated separately with the respective calibration solution. Each probe was dipped into the sample and readings were allowed to stabilize before being recorded for the different parameters. Phosphate was determined by measuring 10 mL of the sample into a 10 mL cuvette and placing it in the cuvette holder of a Hanna HI83399 photometer. The instrument was zeroed followed by the addition of 10 drops of phosphate high range (HR) reagent A and 1 packet of phosphate HR reagent B was added followed by gentle shaking of the cuvette. This mixture was placed in the photometer, allowed to stand for 5 min and phosphate concentration was read off. Nitrate was determined by measuring 10 mL of diluted aliquot (tenfold dilution) of the sample into a 10 mL cuvette and inserting it into the cuvette holder of the photometer followed by pressing the zero button. The cuvette was removed and 1 packet of nitrate reagent was added. The cuvette was capped, shaken vigorously for 10 s, inverted alternately for 50 s and placed into the cuvette holder of the photometer before the nitrate concentration was read off after 4.5 min. Chloride, total nitrogen and total phosphorus were also determined with the same photometer using the methods and reagents outlined in the instruction manual. Biochemical oxygen demand (BOD_5_) after a 5-day incubation period was determined with a modification of the method^56^. Dilution water was prepared by mixing 10 mL of each phosphate buffer, MgSO_4_, CaCl, FeCl_3_, Na_2_SO_3_ and NH_4_Cl with 10 L of distilled water. Two 10 mL aliquots of each effluent were transferred separately into two 300 mL BOD bottle which was then filled up with the dilution water. Two 300 mL BOD bottles were filled with the dilution water and served as blanks. One BOD bottle with effluent and one blank bottle was incubated at 20 °C for 10 days while the dissolved oxygen (DO) content of the effluent in the second BOD bottle (DO_1_) and blank were determined immediately with the Hanna multi-parameter test meter. After 5 days, the DO of the incubated sample (DO_5_) and blank were also determined with the same test meter and BOD_5_ was calculated as:

$$\mathrm{BOD}_5\,(\mathrm{mg L}^{-1})=\frac{(\mathrm{DO}1 -\mathrm{ DO}5) \times \mathrm{ Volume\, of \,BOD\, bottle}}{\mathrm{Volume\,of\, sample}}$$

The Hanna photometer (model: HI83399), Hanna COD medium range (MR) and high range (HR) reagents contained in factory-prepared vials were used for the determination of Chemical Oxygen Demand (COD). The MR and HR reagents were used for determining the COD of samples. Two reagent vials were used for one test; 2 mL (0.2 mL for HR) of deionized water was added to one vial (blank) while 2 mL (0.2 mL for HR) of the sample was added to the second vial. After mixing, the contents were digested in a Hanna reactor (pre-heated to 150 °C) for 2 h and allowed to cool for 20 min. Each vial was inverted 5 times while still warm and allowed to cool to room temperature followed by the zeroing of the Photometer with the blank vial and determination of COD in mg L^−1^ in the sample vial.

### Application of nanoparticles for metal and pesticide removal

The synthesised nanoparticles (10 mg) were separately added to 100 mL aliquots of the wastewater samples and were agitated in a fixed-speed rotator for 180 min at 400 rpm. At the end of the contact time, the samples were filtered through Whatman no. 1 filter paper and analysed with AAS and GC–MS as appropriate. The uptake capacity (mg g^−1^) was determined using the:

1 $$\mathrm{q}_\mathrm{e}=\frac{\mathrm{Co }-\mathrm{ Ce}}{\mathrm{W}} \times \mathrm{ V}$$

where q_e_ represents the uptake capacity of the adsorbents, C_o_ (mg L^−1^) is the initial concentration of analyte in solution, and C_e_ (mg L^−1^) is the residual/equilibrium concentration of the analyte.

### Statistical analysis

Analysis of the physicochemical parameters of each effluent sample was carried out in triplicate. The means and standard deviations of triplicate determinations were calculated and the values obtained were analysed using single-factor analysis of variance (ANOVA). A comparison of means was carried out using Duncan Multiple Range Test. Statistical analysis was carried out with SPSS software (Version 22.0).

### Ethical approval

This experimental research upon plants complies with relevant institutional, national and international guideline and legislation.

## Results and discussion

### Characterisation of nanomaterials

**Figure 2:**
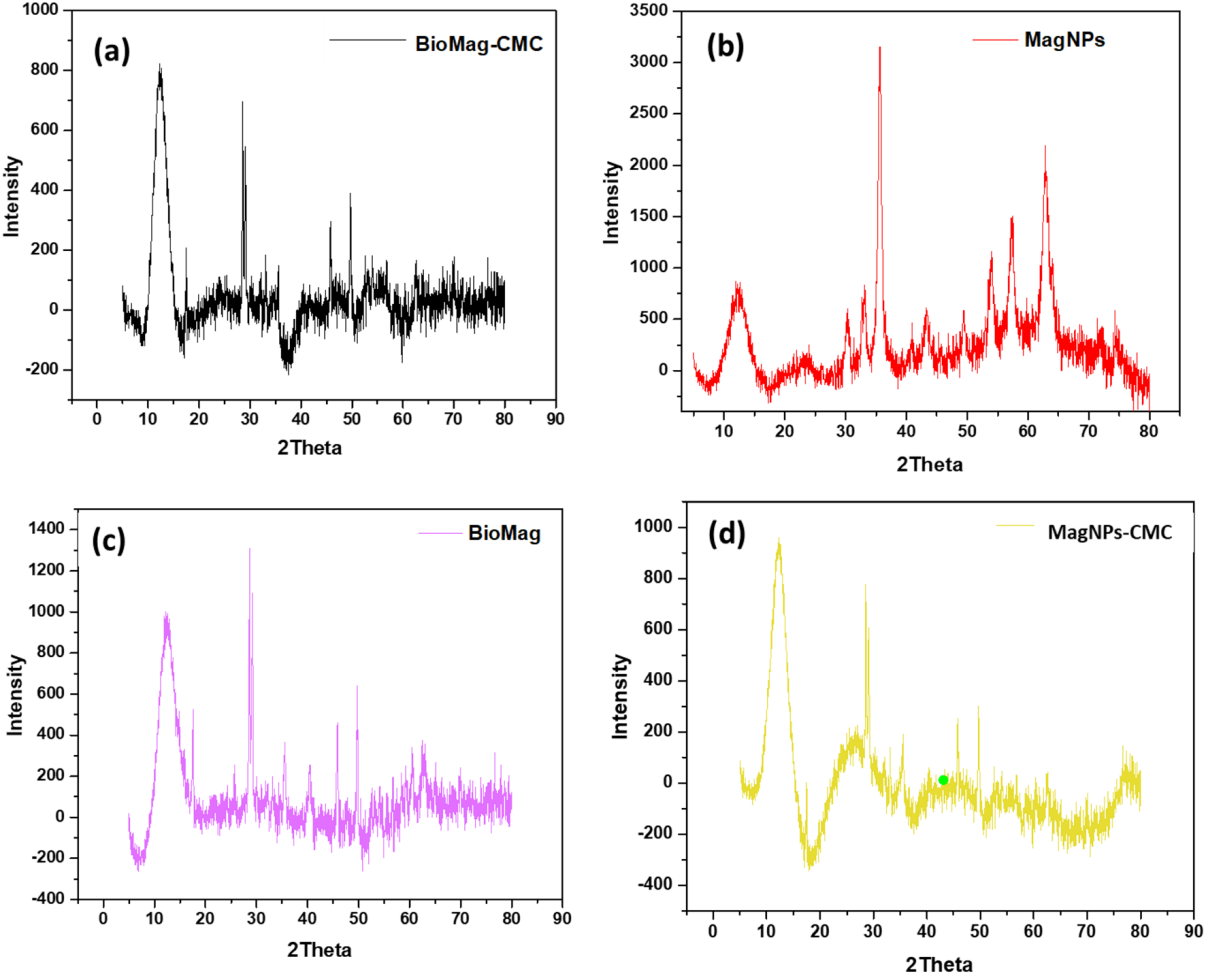
XRD pattern (**a**) BioMag-CMC, (**b**) MagNPs-CMC, (**c**) BioMag and (**d**) MagNPs.

**Figure 3:**
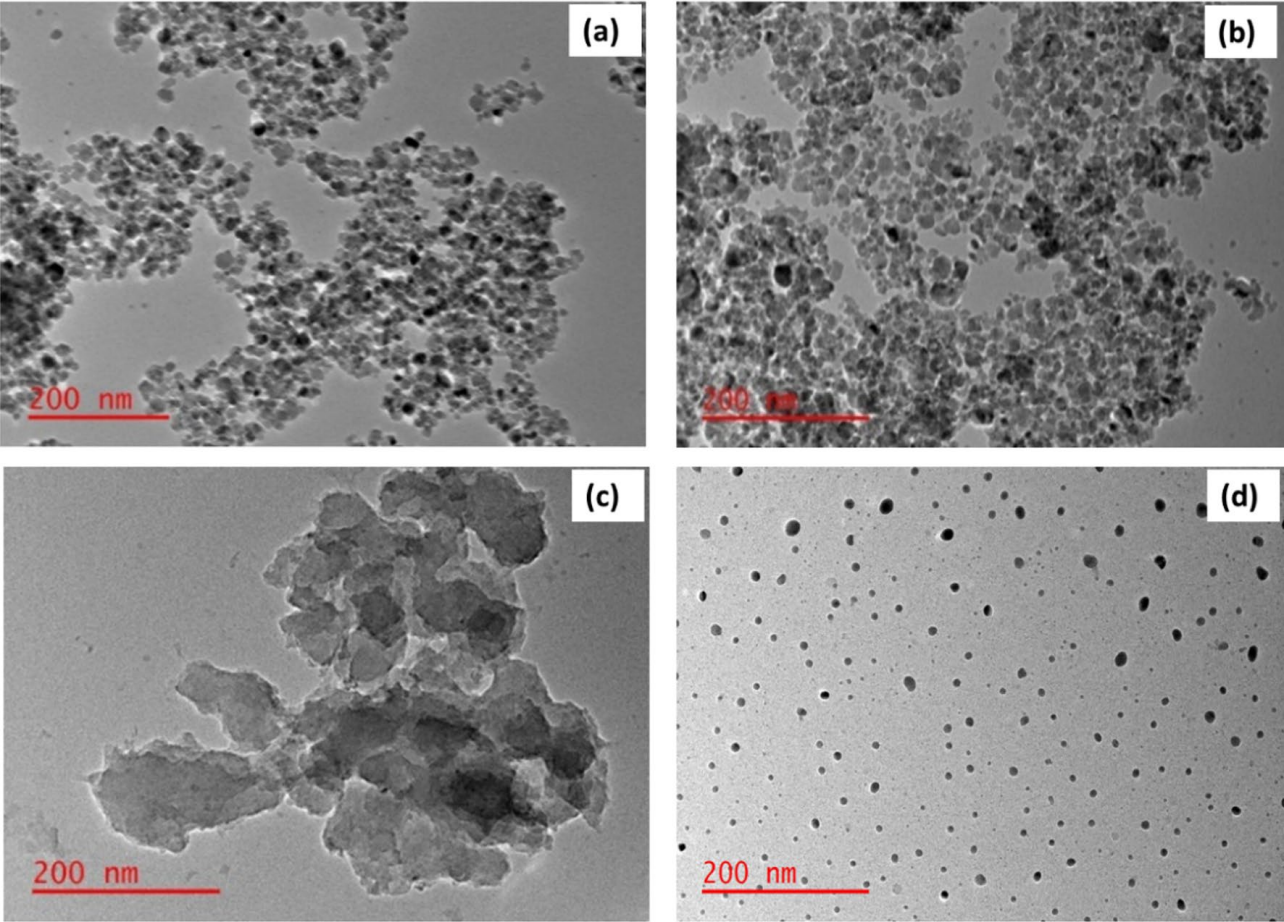
TEM micrograph of (**a**) Biomag-CMC, (**b**) MagNPs-CMC, (**c**) BioMag and (**d**) MagNPs.

**Figure 4:**
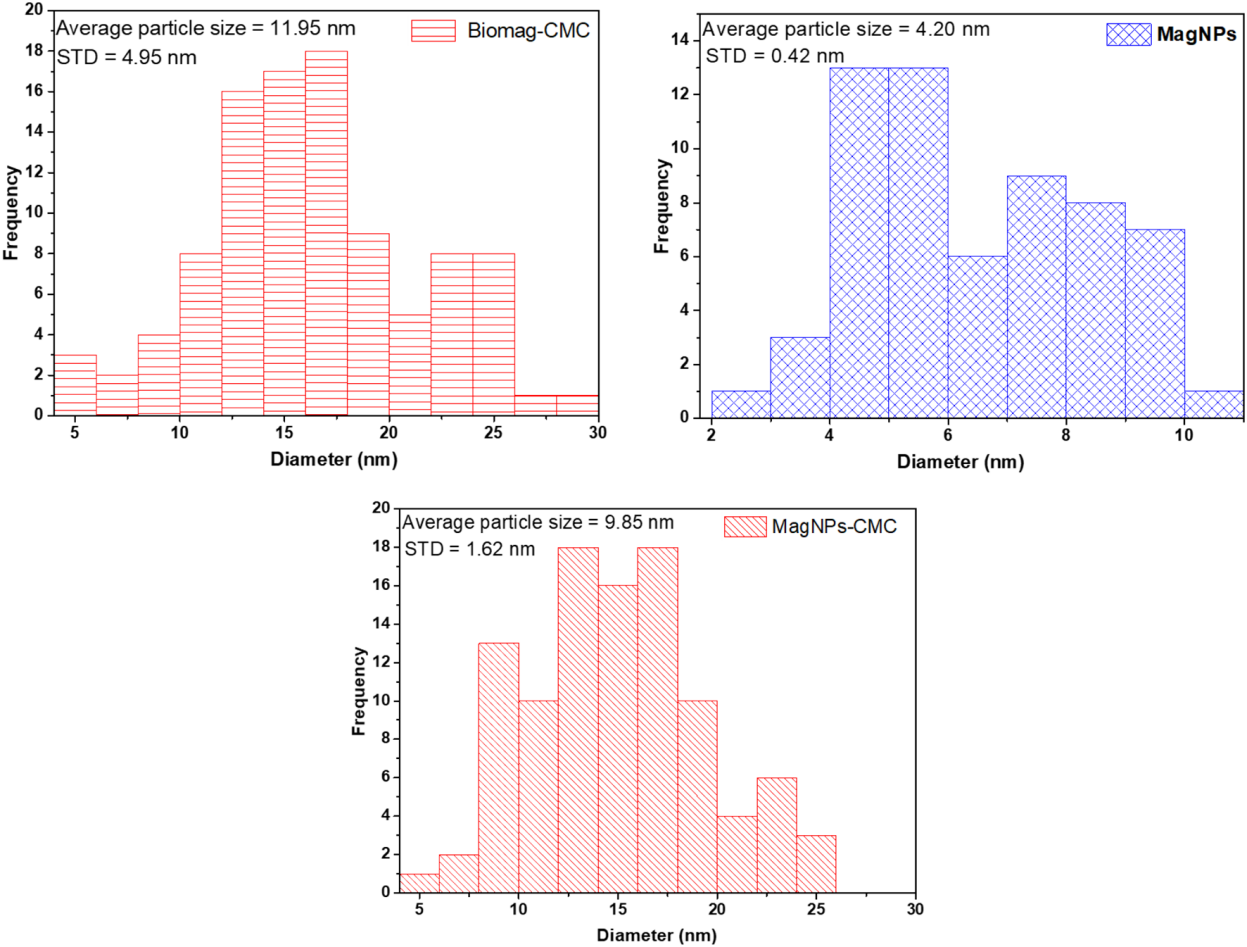
The estimated average particle size of BioMag-CMC, MagNPs and MagNPs-CMC using ImageJ.

The phase and crystallinity of BioMag-CMC, MagNPs-CMC, BioMag and MagNPs were assessed using X-ray diffraction patterns and displayed in Fig. 2. The XRD patterns acquired for MagNPs correspond to JCPDS No.160653. However, the application of a biological route may be responsible for the shift in peaks as observed in the XRD patterns of BioMag-CMC, MagNPs-CMC and BioMag (see Fig. 2). The TEM images for Biomag-CMC, MagNPs-CMC and MagNPs are shown in Fig. 3a,b,d. From the TEM micrograph, well-dispersed spherically shaped NPs with a significantly smaller distribution were observed for MagNPs (see Fig. 3d). The addition of CMC induced agglomeration of spherical NPs acquired for Biomag-CMC and MagNPs-CMC. On the other hand, an irregularly stacked clumps morphology was recorded for BioMag (see Fig. 3c). The average particle sizes of BioMag-CMC, MagNPs and MagNPs-CMC were estimated as 11.95 ± 4.95 nm, 4.20 ± 0.42 nm and 9.85 ± 1.62 nm respectively (see Fig. 4). From the particle size analysis, the inclusion of CMC as an adsorbent modifier was noticed to enhance the particle size distribution as observed with the nanoparticles of the magnetite.

**Figure 5:**
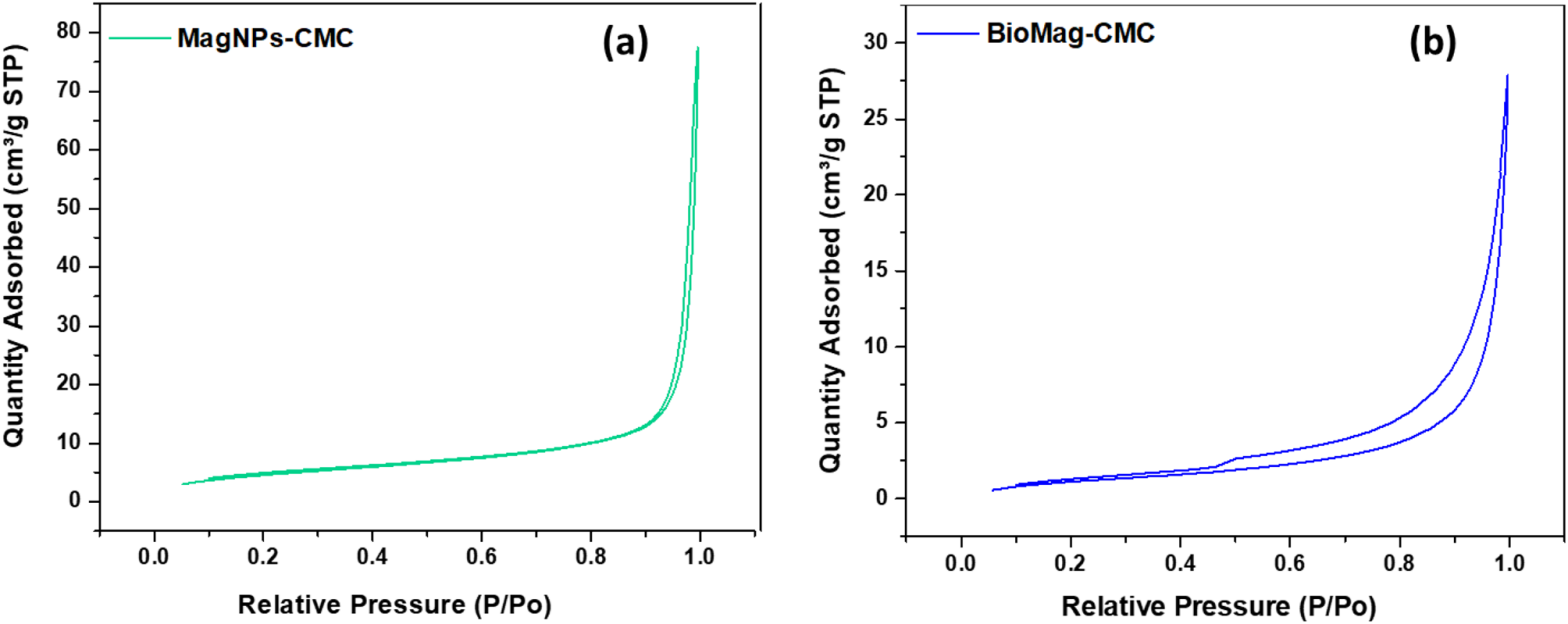
The nitrogen adsorption–desorption curves of (**a**) MagNPs-CMC, (**b**) BioMag-CMC.

The specific surface area and pore volume of BioMag-CMC and MagNPs-CMC were assessed using the BET nitrogen adsorption–desorption technique. These physical parameters increased in the order BioMag-CMC > MagNPs-CMC (see Table 2). Meanwhile, the isotherms profile for BioMag-CMC and MagNPs-CMC exhibited a characteristic type-IV curve with a hysteresis loop within a relative pressure (P/P0) > 0.45 and > 0.9 (see Fig. 5). This could be credited to the fact that the capillary condensation and evaporation occurred at different pressure. To exhibit a type-IV isotherm profile shows that the material (BioMag-CMC and MagNPs-CMC) under investigation sustained mesoporous characteristics with a pore diameter in the range of 2–50 nm and this is in close agreement with the values obtained from TEM measurement. On the other hand, the pore diameter of BioMag-CMC and MagNPs-CMC was assessed by making use of the Barrett–Joyner–Halenda (BJH) theory (see Table 2).

**Table 2:** Textural properties of MagNPs-CMC and BioMag-CMC.

Adsorbents	Surface area/m^2^ g^−1^	Pore volume/cm^3^ g^−1^	Pore diameter/nm
MagNPs-CMC	16.024	0.65000	23.3408
BioMag-CMC	4.7615	0.038493	24.1725

**Figure 6:**
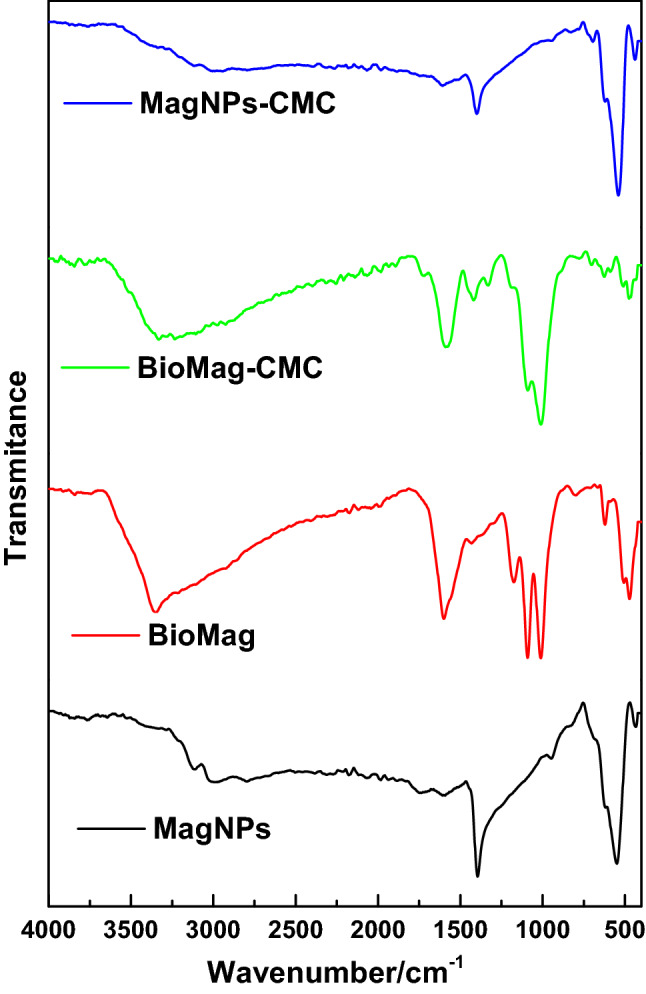
The FTIR spectra of BioMag, MagNPs, BioMag-CMC and MagNPs-CMC.

The spectra acquired for BioMag, MagNPs, BioMag-CMC and MagNPs-CMC showed close similarities in their bands with a slight variance in the intensity of their peaks (see Fig. 6). The broad peak between 3350 and 3450 cm^−1^ may be attributed to the hydroxyl (–CHOH) on the surface of the nanoparticles. The sharp peak at 1608–1612 cm^−1^ and 1031–1033 cm^−1^ are assigned to the stretching vibrations of the C=O and –OH groups, respectively^57–61^. The modification of the magnetite was observed to cause a shift in bands and a slight adjustment in the peak intensities. The implication of using CMC as a modifier was observed on the spectra of BioMag-CMC and MagNPs-CMC having enhanced broad peaks between 3350 and 3450 cm^−1^ (see Fig. 6).

### Physicochemical parameters of the raw effluents

The mean values for the physicochemical parameters of the 13 raw effluent samples are shown in Tables S1–S4. Biochemical oxygen demand (BOD) describes the amount of oxygen needed by microorganisms to biodegrade organic matter. Meanwhile, a high level of BOD indicates a high discharge of biodegradable waste into effluents. The assessment of BOD in the industrial effluents was performed and the results reflect a low level of BOD (see Table 3). Chemical oxygen demand describes the amount of oxidant needed to break down inorganic and organic matter. As displayed in Table 4, the studied effluents were noticed to be higher than the recommended maximum concentration (RMC) by USEPA with the exception of SUWK, PTWK, PTK, CMTWM and CTWA. PCUWA was observed to have the highest concentration of COD and this could be attributed to a high amount of oxidant used in the production of some personal care products. However, MagNPs-CMC demonstrated a 62.48% reduction of COD when applied to PCUWA (see Table 4). TUKW was the most acidic with a pH of 3.73 ± 0.05 while FTWR and FCTWE had similar basic pH values which were significantly higher (P < 0.05) than pH values acquired for other effluents. The variation in effluent pH is influenced by the quality of the industrial activity. Solution pH can influence the availability of micronutrients and may pose danger to aquatic life.

**Table 3:** Ranges of BOD analysis obtained before and after remediation for the 13 study sites.

Effluent	BOD_5_ of raw WW (mg L^−1^)	BOD_5_ of WW samples after application of nanoparticles (mg L^−1^)			
		MagNPs	BioMag	MagNPs-CMC	BioMag-CMC
PCUWA	23.4	61.5	16.8	20.1	21.0
TUWK	9	57.3	32.7	21.6	39.9
SUWK	40.5	2.1	8.7	21.3	42.3
MUWA	6.3	103.8	99.9	7.2	42.3
PTWK	1.5	30.3	54.6	8.4	16.2
TUKW	14.1	18	26.4	21	30.9
BTWR	32.1	62.1	56.4	46.2	39
PUWA	26.4	49.2	57.9	48.9	26.1
PTK	5.1	3.3	1.8	7.2	21
FTWR	16.8	73.2	17.1	41.1	22.8
FCTWE	13.2	13.5	21.6	41.1	15.6
CMTWM	24.9	70.8	28.5	28.5	40.2
CTWA	42.9	62.4	58.2	37.5	48.6

**Table 4:** Ranges of COD analysis obtained at the 13 study sites with their percentage reductions.

Effluent	Original WW COD	COD after filtration	COD after application of Nanomaterials and filtration				% COD reduction			
			BioMag-CMC	MagNPs-CMC	BioMag	MagNPs	BioMag-CMC	MagNPs-CMC	BioMag	MagNPs
PCUWA	7271	7020	3558	2702	3370	3118	51.07	62.84	53.66	57.12
TUWK	3712	3698	2015	1325	1947	2315	45.71	64.3	47.55	37.63
SUWK	257	212	105	95	154	128	59.04	63.04	40.09	50.21
MUWA	1570	1450	498	462	415	526	68.29	70.52	73.57	66.5
PTWK	166	157	116	93	104	142	30.12	43.97	37.35	14.46
TUKW	4635	4619	2335	2190	2295	2537	49.62	52.75	50.49	45.26
BTWR	6244	5860	3173	2482	2218	2593	49.18	60.25	64.48	58.47
PUWA	5909	5734	2311	2137	3018	3570	60.89	63.83	48.93	39.58
PTK	969	886	316	341	488	380	67.37	64.82	49.66	60.8
FTWR	6433	6372	2614	3016	2950	3694	59.37	53.12	54.14	42.58
FCTWE	2313	2247	810	789	850	753	64.98	65.9	63.26	67.46
CMTWM	170	162	97	69	105	111	42.94	59.41	38.24	34.71
CTWA	610	595	215	187	340	301	64.75	68.34	44.26	50.66

As shown in Tables S1 and S2, electrical conductivity (7049.55 ± 16.26 µS cm^−1^) and TDS (3614.66 ± 126.57 mg L^−1^) values of FTWR were significantly higher (P < 0.05) than those of other effluents (see Supplementary information). Meanwhile, after the remediation step, PUWA was noticed to have the lowest mean value for electrical conductivity, EC (99.18 ± 0.44 µS cm^−1^) and TDS (99.18 ± 0.44). On the other hand, the electrical conductivity (EC) of TUWK, SUWK, MUWA, PTWK, TUKW, BTWR, PUWA and FTWR were higher than the USEPA’s RMC of 2500 µS cm^−1^. This could be attributed to a high amount of dissolved ions from decomposed materials (see Table S7) (Supplementary information). However, nitrate (83.91 ± 6.77 mg L^−1^) and total nitrogen (127.04 ± 10.55 mg L^−1^) of treated PUWA were significantly higher (P < 0.05) compared to values of the same parameters for the other effluents (see Table S4). Phosphate concentrations in the MUWA and BTWR were similar (P > 0.05) but the lowest concentrations were recorded in the TUWK and CTWA. On the other hand, a total P concentration of 0.32 ± 0.07 mg L^−1^ was recorded for SUWK while the highest significant (P < 0.05) concentration of 27.11 ± 3.84 mg L^−1^ was recorded for BTWR.

The high value of sulphate could be attributed to a high volume of detergents used during clean-up operations in the brewery. However, high levels of nitrates and phosphate may boost the growth of vegetation in the aquatic ecosystem and can directly increase oxygen demand. Meanwhile, the mean concentrations of chloride (56.12 ± 4.84) and sulphate (505.61 ± 27.40) were significantly higher (P < 0.05) in TUWK and FCTWE respectively. However, the concentration of chloride for all the effluents was below the RMC by USEPA (see Tables S3 and S7). A similar trend with the exception of PTWK, TUKW and TUWK was noticed for sulphate (see Table S3) (see Supplementary information). Finally, EC, pH and TDS of PCUWA, TUWK, SUWK, MUWA, PTWK, TUKW, BTWR, PUWA, PTK, FTWR, FCTWE, CMTWM and CTWA were significantly reduced after treatment with MagNPs, BioMag, MagNPs-CMC and BioMag-CMC as shown in Tables S1 and S2 (see Supplementary information). Hence, effective pre-treatment of effluent can ensure a safe aquatic ecosystem for man.

### Assessment of pesticides and heavy metal ions in industrial wastewater samples

The concentration of heavy metals in industrial wastewater samples collected from five states in Nigeria was quantified and presented in Table 2. All the targeted heavy metals (Cd, Cr and Pb) were significantly detected in all the effluent samples. The concentration of Cd ranged from 0.008 ± 0.003 mg L^−1^ for samples SUWK and PTK to 0.056 ± 0.010 mg L^−1^ for PUWA. Cadmium concentrations in the samples collected from 13 different industries across five states in Nigeria were in the order SUWK = PTK > TUKW > PTWK > MUWA > FTWR > TUWK > BTWR > FCTWE > CMTWM > PCUWA > CTWA > PUWA. All samples reported in this study were higher than WHO’s RMC of 0.003 mg L^−1^^62^. The high level of Cd in some of the samples was expected but estimating a high concentration of Cd in SUWK, MUWA, BTWR and PTK were not expected because these companies deal mainly with foods and drugs (see Table 5). Hence, it is imperative to identify and eliminate cadmium-leaching materials from the vicinity and processes of these industries. This hazardous metal ion commonly finds its way into the water bodies via fertilizer runoff from farmlands, waste batteries, paints, alloys, coal combustion, printing, pulp, refineries, steel smelting and electroplating industries^63^. Different sicknesses caused by medium and acute cadmium exposure include hypertension, renal damage, liver and kidney damage, lung inefficiency, initiation of cancer growth and calcium depletion in bones^64,65^. These concentrations were lower than the levels of Cd (0.065 ± 0.001 mg L^−1^) as reported by Bawa‑Allah^66^ and higher than the concentration of Cd (0.12 mg L^−1^) that was reported by Agoro^67^.

**Table 5:** Concentrations of the metals in the wastewater after remediation with the synthesized nanomaterials.

Wastewaters	Initial concentrations (mg L^−1^)			Nanoparticles and metal removal (mg L^−1^)											
				BioMag-CMC			MagNPs-CMC			BioMag			MagNPs		
	Cd	Cr	Pb	Cd	Cr	Pb	Cd	Cr	Pb	Cd	Cr	Pb	Cd	Cr	Pb
PCUWA	0.052 ± 0.021^a^	0.024 ± 0.008^a^	0.026 ± 0.011^a^	0.003	0.005	0.0008	0.012	0.005	0.0001	0.009	0.007	0.0005	0.006	0.003	0.0011
TUWK	0.022 ± 0.015^b^	0.033 ± 0.016^a^	0.019 ± 0.008^a^	0.013	0.004	0.0007	0.015	0.008	0.0009	0.003	0.004	0.0010	0.016	0.002	0.0013
SUWK	0.008 ± 0.003^c^	0.012 ± 0.005^b^	0.009 ± 0.007^b^	0.006	0.008	0.0014	0.003	0.007	0.0011	0.002	0.009	0.0006	0.004	0.007	0.0006
MUWA	0.013 ± 0.006^c^	0.011 ± 0.003^b^	0.016 ± 0.012^a^	0.012	0.006	0.0011	0.013	0.011	0.0009	0.011	0.002	0.0014	0.010	0.008	0.0018
PTWK	0.011 ± 0.002^c^	0.040 ± 0.016^c^	0.019 ± 0.008^a^	0.008	0.010	0.0009	0.005	0.008	0.0011	0.007	0.014	0.0016	0.006	0.010	0.0008
TUKW	0.009 ± 0.004^c^	0.039 ± 0.009^c^	0.023 ± 0.011^a^	0.005	0.012	0.0060	0.003	0.009	0.0016	0.002	0.012	0.0039	0.004	0.018	0.0022
BTWR	0.028 ± 0.013^b^	0.022 ± 0.010^a^	0.049 ± 0.027^c^	0.010	0.006	0.0005	0.008	0.007	0.0008	0.005	0.011	0.0011	0.005	0.010	0.0008
PUWA	0.056 ± 0.010^a^	0.015 ± 0.008^a^	0.012 ± 0.006^b^	0.009	0.001	0.0010	0.004	0.009	0.0012	0.015	0.011	0.0011	0.010	0.004	0.0016
PTK	0.008 ± 0.003^c^	0.027 ± 0.021^a^	0.015 ± 0.008^a^	0.007	0.009	0.0023	0.009	0.003	0.0015	0.005	0.006	0.0024	0.004	0.007	0.0022
FTWR	0.021 ± 0.009^b^	0.018 ± 0.009^b^	0.034 ± 0.013^a^	0.004	0.009	0.0010	0.003	0.004	0.0015	0.003	0.002	0.0009	0.009	0.002	0.0012
FCTWE	0.035 ± 0.012^d^	0.012 ± 0.004^b^	0.029 ± 0.015^a^	0.011	0.005	0.0007	0.004	0.009	0.0008	0.009	0.002	0.0010	0.007	0.007	0.0005
CMTWM	0.035 ± 0.019^d^	0.635 ± 0.240^d^	2.215 ± 0.841^c^	0.005	0.007	0.0006	0.011	0.009	0.0010	0.008	0.007	0.0009	0.006	0.004	0.0007
CTWA	0.053 ± 0.028^a^	0.616 ± 0.095^d^	1.587 ± 0.603^c^	0.006	0.005	0.0012	0.004	0.008	0.0004	0.005	0.008	0.0014	0.002	0.004	0.0005

The concentrations of Cr ranged from 0.011 ± 0.003 mg L^−1^ for sample MUWA to 0.635 ± 0.240 mg L^−1^ for CMTWM. The elevated concentrations observed were recorded in the order MUWA > SUWK > FCTWE > PUWA > FTWR > BTWR > PCUWA > PTK > TUWK > TUKW > PTWK > CTWA > CMTWM for the samples investigated. The highest level of Cr was recorded in sample CMTWM. Meanwhile, the concentration of Cr estimated for the 13 samples was noticed to be higher than the RMC of 0.01 mg L^−1^ and 0.015 mg L^−1^ as given by WHO and USEPA in surface water and effluent water, respectively. High exposure to Cr may lead to severe effects such as perforation of the nasal septum, asthma, bronchitis, pneumonitis, inflammation of the larynx and liver, and increased occurrence of bronchogenic carcinoma^68,69^. On the other hand, skin contact with chromium compounds have been associated with some skin problems, such as skin allergies, dermal necrosis, dermatitis, and dermal decay^70^. Hence, it is important to devise an effective means of eliminating this recalcitrant water contaminant.

The observed Pb concentrations in the samples collected from five states were noticed to range from 0.009 ± 0.007 for SUWK to 2.215 ± 0.841 for CMTWM. Meanwhile, the elevated concentrations observed were recorded in the order SUWK > PUWA > PTK > MUWA > TUWK > PTWK > TUKW > PCUWA > FCTWE > FTWR > BTWR > CTWA > CMTWM. With the exception of SUWK, the concentrations of Pb in the samples were found to be higher than the RMC of 0.01 mg L^−1^ and 0.015 mg L^−1^ as given by WHO and USEPA in surface water and effluent water, respectively. The main route of Pb in wastewater is runoff from mining, leather tanning, metal processing and electroplating industries. Meanwhile, lead toxicity might pose a minor or major health challenge as it has been reported to cause learning and behavioural difficulty in children, malaise, loss of appetite, anaemia and organ failure^71–73^.

The evaluation step revealed a significant amount of metal ion contaminants (Cd, Cr and Pb) in the industrial effluent collected from five states. Hence, it is imperative to eliminate these water contaminants from the water bodies or reduce the level of these toxic metal ions to RMC using a cheap and effective removal technology. The application of Biomag, MagNPs, Biomag-CMC and MagNPs-CMC as adsorbent for the removal of Cd, Cr and Pb from industrial effluent (PCUWA, TUWK, SUWK, MUWA, PTWK, TUKW, BTWR, PUWA, PTK, FTWR, FCTWE, CMTWM and CTWA) was observed to be effective in the remediation step. The adsorbent demonstrated an affinity for the contaminant in the order Pb > Cr > Cd (see Table 5). The residual concentration of the contaminants was close to the RMC as given by WHO and USEPA after the sorbent-sorbate interaction. To further understand the effectiveness of the synthesised nanocomposites and nanoparticles, the uptake capacities of these materials were estimated and presented in Table S5 (see Supplementary information). The average uptake capacities of Biomag-CMC, Biomag, MagNPs and MagNPs-CMC, are 0.180 ± 0.015, 0.180 ± 0.016, 0.176 ± 0.016 and 0.173 ± 0.029, respectively. Hence, BioMag-CMC has demonstrated superior potential to sequester metal ions from industrial wastewater regardless of the interference from other analytes. The FTIR assessment of BioMag-CMC, BioMag, MagNPs and MagNPs-CMC revealed the presence of functional groups (–OH, –NH and C=O) that have the capacity to trap metal ions via ion exchange or electrostatic interactions.

Table 6 displays the level of pesticide residues in the PTWK wastewater sample. In the raw pesticide wastewater, dieldrin (1.76 ng L^−1^) and endrin (0.89 ng L^−1^) were noticed to have the highest and least concentration. This could be attributed to the kind of activities within the industry. A relatively low level of pesticides was detected in the wastewater sample and this could be associated with the hydrophobic characteristics of these analytes. Trace or ultra-trace amounts of these analytes can pose a danger to both aquatic organisms and man. A significant amount of chlordane, endosulfan, DDT, DDE and heptachlor were detected in the sample. Meanwhile, the aforementioned analytes possess the capacity to negatively impact the biodiversity of the aquatic ecosystem. This is attributed to the endocrine and estrogenic disrupting properties of these pesticides^74^. Hence, it is imperative to pre-treat these industrial effluents before discharge. The residual concentrations of the pesticides were evaluated after treatment with BioMag, MagNPs, BioMag-CMC and MagNPs-CMC nanocomposites and results are presented in Table 6. Meanwhile, the uptake capacity of the adsorbent was assessed and reported in Table S6 (see supplementary information). The nanocomposite materials were observed to effectively reduce the level of pesticides via the batch adsorption technique. MagNPs-CMC with an average removal capacity of 11.6 ± 8.202 mg g^−1^ was observed to reduce the concentration of β-BHC, heptachlor, α-chlordane, endosulfan I, P,P′-DDD, endosulfan II, P,P′-DDT, endrin aldehyde, endosulfan sulphate, methoxychlor and nndrin ketone to a level of no detection. The uptake potential of these materials could be due to the characteristic nature (hydrophobicity and electrostatic interaction) of the nanocomposite. Finally, uptake capacities of BioMag-CMC and MagNPs-CMC in the removal of OCPs and heavy metals were > 68% after the fifth cycle.

**Table 6:** Initial and final concentrations of residues in pesticide wastewater.

SN	Pesticide residues	Initial concentrations (ng L^−1^)	Concentrations at equilibrium (ng L^−1^)			
			Biomag	MagNPs	Biomag-CMC	MagNPs-CMC
1	α-BHC	1.10	0.05	ND	ND	0.52
2	β-BHC	0.98	0.59	–	0.05	ND
3	γ-BHC	1.01	0.65	ND	0.09	0.17
4	Heptachlor	0.97	0.81	1.01	0.29	0.30
5	δ-BHC	0.93	0.30	0.17	0.70	0.16
6	Aldrin	1.03	0.54	0.47	0.14	0.38
7	Heptachlor epoxide	0.99	ND	0.33	ND	ND
8	γ-Chlordane	1.07	0.20	ND	0.85	0.66
9	α-Chlordane	0.98	ND	0.67	0.99	ND
10	Endosulfan I	1.28	0.20	0.21	ND	ND
11	P,p′-DDE	1.16	0.17	0.35	1.18	0.04
12	Dieldrin	1.76	0.40	0.08	0.70	0.14
13	Endrin	0.89	ND	1.30	0.61	0.30
14	P,P′-DDD	0.98	0.18	ND	0.17	ND
15	Endosulfan II	1.21	ND	ND	ND	ND
16	P,P′-DDT	1.37	ND	ND	0.18	ND
17	Endrin aldehyde	1.20	ND	ND	0.08	ND
18	Endosulfan sulphate	1.11	ND	ND	0.90	ND
19	Methoxychlor	0.90	ND	ND	ND	ND
20	Endrin ketone	1.74	ND	ND	ND	ND

*ND* not detected.

## Conclusion

The concentrations of pesticides and metal ions were evaluated in the industrial wastewater collected from five states in Nigeria. The level of these analytes in the samples is a function of the quality of industrial activities and the waste management systems of these industries. SUWK and PTK contained the least level of metal ions (Cd, 0.008 ± 0.003 mg L^−1^), while, the highest concentration of metal ions was found in CMTWM (Pb, 2.215 ± 0.841 mg L^−1^). A significant amount of OCPs was recorded in the samples collected from pesticide industrial effluent (PTWK). The concentration of the OCPs ranged from 1.76 ng L^−1^ (Dieldrin) to 0.89 ng L^−1^ (endrin). The levels of contaminants in most of the samples exceeded the recommended maximum concentrations by the stakeholders. Thus, the presence of heavy metal ions and pesticide residues in the industrial effluents may pose potential hazards to aquatic organisms and man if they find their way into the surrounding water bodies. The application of BioMag, MagNPs, BioMag-CMC and MagNPs-CMC as adsorbents was effective in pollutants removal. MagNPs-CMC and BioMag-CMC with average uptake capacity of 11.6 ± 8.202 mg g^−1^ and 0.181 ± 0.015 mg g^−1^ demonstrated outstanding potential in pesticide and heavy metal removal, respectively. The nanomaterials were observed to effectively reduce the physicochemical property (pH, EC and TDS) of the effluents. Hence, the removal process of contaminants and other unidentified dissolved solids/ions from wastewater will positively impact the biochemical characteristics of wastewater and NPs. Finally, the routine monitoring and pre-treatment of industrial effluents to prevent OCP and heavy metal ions contamination are essential for the sustainability of a clean and healthy aquatic ecosystem.

## Supplementary Information

Supplementary Information.

## Data Availability

The authors declare that all data generated and analysed are available within the manuscript [and its supplementary information file].
