# Supplementary material for: Evaluation and remediation protocol of selected organochlorine pesticides and heavy metals in industrial wastewater using nanoparticles (NPs) in Nigeria
Source: Sci Rep. 2023 Feb 7;13:2170. doi: 10.1038/s41598-023-28761-3 (PMC9905072; doi:10.1038/s41598-023-28761-3)
Supplement: Supplementary file 1 — Supplementary Information. [file 41598_2023_28761_MOESM1_ESM.docx]

**Evaluation and remediation protocol of selected organochlorine pesticides and heavy metal**

**Jude Chidozie Nnaji^1^, James Friday Amaku^1^*, Okoche Kelvin Amadi^1^ & Solomon Ireji Nwadinobi^1^**

^1^Department of Chemistry, Michael Okpara University of Agriculture Umudike, P.M.B 7267 Umuahia, Abia State, Nigeria

*corresponding author: [amakufj2006@gmail.com](mailto:amakufj2006@gmail.com)

| **Effluent** | pH | MagNPs | BioMag | MagNPs-CMC | BioMag-CMC | EC (µS/cm) | MagNPs | BioMag | MagNPsCMC | BioMag-CMC |
| --- | --- | --- | --- | --- | --- | --- | --- | --- | --- | --- |
| **PCUWA** | 7.22±0.23 | 6.56 | 5.22 | 6.54 | 5.6 | 1065.71±14.85 | 1087 | 1374 | 1611 | 1285 |
| **TUWK** | 9.16±0.02 | 8.46 | 8.32 | 8.5 | 8.3 | 696.37±1.41 | 717 | 953 | 1115 | 819 |
| **SUWK** | 9.24±0.06 | 8.56 | 7.37 | 7.92 | 7.13 | 465.05±8.49 | 495 | 708 | 989 | 614 |
| **MUWA** | 4.66±0.04 | 4.99 | 4.13 | 4.47 | 2.64 | 431.58±2.12 | 416 | 850 | 979 | 1104 |
| **PTWK** | 5.90±0.13 | 7.25 | 5.55 | 6.31 | 3.87 | 343.65±7.07 | 390 | 794 | 829 | 603 |
| **TUKW** | 3.73±0.05 | 3.65 | 3.3 | 3.41 | 3.25 | 3175.82±23.08 | 3220 | 3480 | 3684 | 3222 |
| **BTWR** | 9.25±0.85 | 10.02 | 8.34 | 8.89 | 8.03 | 3142.71±4.24 | 1080 | 1001 | 1053 | 869 |
| **PUWA** | 8.59±0.71 | 8.21 | 7.01 | 7.92 | 8.1 | 847.90±10.61 | 724 | 1099 | 1202 | 887 |
| **PTK** | 4.61±0.03 | 4.74 | 4.09 | 4.47 | 3.76 | 222.93±0.57 | 270 | 637 | 795 | 420 |
| **FTWR** | 11.45±0.31 | 11.3 | 11.2 | 11.26 | 10.29 | 7049.55±16.26 | 6334 | 12730 | 12190 | 16660 |
| **FCTWE** | 11.33±0.10 | 10.16 | 8.55 | 10.03 | 8.97 | 1878.20±27.58 | 1519 | 1568 | 1539 | 1409 |
| **CMTWM** | 8.61±0.10 | 6.29 | 4.4 | 6.33 | 3.43 | 434.60±4.24 | 451 | 844 | 863 | 813 |
| **CTWA** | 8.94±0.11 | 8.12 | 6.05 | 7.46 | 4.35 | 219.59±14.14 | 276 | 691 | 660 | 588 |

**Table S1**. Solution pH and electrical conductivity (EC) of industrial effluents.

|  | TDS (mg/L) | MagNPs | BioMag | MagNPs-CMC | BioMag-CMC | | Nitrate (mg/L) | MagNPs | BioMag | MagNPs-CMC | BioMag-CMC |
| --- | --- | --- | --- | --- | --- | --- | --- | --- | --- | --- | --- |
| **PCUWA** | 532.71±7.78 | 527 | 680 | 805 | 644 | PCUWA | 46.77±3.16 | 51.7 | 46.4 | 21.3 | 39.8 |
| **TUWK** | 348.26±0.50 | 358 | 462 | 557 | 409 | TUWK | 4.11±0.75 | 2.2 | 5.8 | 6.9 | 2.6 |
| **SUWK** | 228.50±9.19 | 247 | 352 | 489 | 307 | SUWK | 15.10±1.81 | 10.4 | 14.7 | 5.9 | 17.6 |
| **MUWA** | 215.41±1.08 | 211 | 425 | 489 | 556 | MUWA | 14.95±1.22 | 3.3 | 12.6 | 8.7 | 11.2 |
| **PTWK** | 171.49±3.54 | 195 | 397 | 414 | 301 | PTWK | 3.93±0.46 | 1.8 | 5.1 | 3.9 | 5.3 |
| **TUKW** | 1587.50±12.02 | 1607 | 1738 | 1842 | 1566 | TUKW | 8.37±0.94 | 5.2 | 8.9 | 7.5 | 8.8 |
| **BTWR** | 1560.48±15.56 | 940 | 500 | 526 | 435 | BTWR | 4.65±0.87 | 4.1 | 5.1 | 6.2 | 5.4 |
| **PUWA** | 423.75±4.95 | 363 | 550 | 599 | 443 | PUWA | 57.42±6.29 | 37.3 | 55.3 | 50.5 | 61.7 |
| **PTK** | 111.58±0.51 | 135 | 318 | 398 | 209 | PTK | 10.19±2.44 | 6.6 | 11.9 | 10.3 | 2.4 |
| **FTWR** | 3614.66±126.57 | 3017 | 7877 | 6359 | 8324 | FTWR | 10.57±1.24 | 6.5 | 12.9 | 10.4 | 14.2 |
| **FCTWE** | 939.50±13.43 | 719 | 770 | 660 | 760 | FCTWE | 7.67±0.94 | 7.8 | 8.5 | 7.9 | 8.3 |
| **CMTWM** | 216.35±1.41 | 225 | 432 | 429 | 406 | CMTWM | 1.64±0.07 | 1.2 | 2.8 | 2.1 | 1.8 |
| **CTWA** | 108.29±7.78 | 138 | 346 | 330 | 295 | CTWA | 18.66±1.90 | 13.6 | 19.5 | 17.3 | 19.7 |

**Table S2**. Total dissolved solids (TDS) and nitrate content of of industrial effluents.

**Table S3**. Total phosphate (P), chloride (Cl) and sulphate (SO_4_^2-^) content of raw and nanocomposite treated effluents

| Effluent | Total P (mg/L) | MagNPs | BioMag | MagNPs-CMC | BioMag-CMC | **Cl- (mg/L**) | MagNPs | BioMag | MagNPs-CMC | BioMag-CMC | **SO_4_^2-^(mg/L)** | MagNPs | BioMag | MagNPs-CMC | BioMag-CMC |
| --- | --- | --- | --- | --- | --- | --- | --- | --- | --- | --- | --- | --- | --- | --- | --- |
| PCUWA | 20.1±3.41 | 12.71 | 5.03 | 10.26 | 9.11 | 17.73±0.96 | 15.2 | 15.3 | 7.6 | 4.8 | 71.08±10.32 | 60 | 28 | 54 | 31 |
| TUWK | 5.94±0.71 | 4.18 | 3.64 | 4.96 | 4.06 | 56.12±4.84 | 35.1 | 8.6 | 17.3 | 23.8 | 43.10±1.95 | 7 | 10 | 13 | 25 |
| SUWK | 1.14±0.20 | 0.74 | 0.55 | 0.47 | 0.41 | 13.25±1.88 | 10.5 | 3.4 | 17 | 7.6 | 1.20±0.08 | 1 | 1 | 1 | 1 |
| MUWA | 9.41±0.65 | 2.43 | 1.05 | 7.4 | 1.45 | 18.42±1.59 | 15.2 | 3.3 | 12.4 | 5.7 | 240.53±11.20 | 4 | 8 | 4 | 1 |
| PTWK | 2.64±0.39 | 1.99 | 1.24 | 2.6 | 1.4 | 40.20±2.71 | 28.1 | 15.1 | 20.7 | 18.6 | 6.02±0.71 | 2.1 | 4.1 | 2.6 | 2.3 |
| TUKW | 9.77±1.28 | 2.16 | 1.67 | 3.06 | 4.74 | 51.33±6.29 | 29.6 | 11.4 | 16.9 | 20.6 | 72.55±3.04 | 140 | 168 | 125 | 139 |
| BTWR | 27.11±3.84 | 11.19 | 7.93 | 9.47 | 8.55 | 4.10±0.62 | 12.2 | 1.9 | 0.8 | 0.5 | 160.70±11.14 | 34.2 | 28.11 | 30.47 | 26.39 |
| PUWA | 11.30±2.07 | 0.62 | 8.33 | 7.41 | 1.13 | 20.76±5.11 | 14.5 | 3.7 | 5.7 | 6.9 | 37.81±1.22 | 7.41 | 8.33 | 9.45 | 12.77 |
| PTK | 2.80±0.63 | 0.44 | 0.22 | 0.19 | 0.32 | 1.44±0.27 | 0.8 | 0.5 | 0.5 | 0.3 | 13.06±0.08 | 10 | 4 | 10 | 11 |
| FTWR | 4.46±1.29 | 3.57 | 0.82 | 0.98 | 1.32 | 11.27±0.85 | 9.4 | 2.9 | 8.1 | 3.4 | 390.14±12.88 | 37 | 30 | 31 | 43 |
| FCTWE | 1.21±0.02 | 0.43 | 0.67 | 0.38 | 0.75 | 25.71±3.65 | 20.6 | 5.9 | 14.5 | 6.9 | 505.61±27.40 | 66.91 | 87.1 | 80.47 | 101.65 |
| CMTWM | 0.57±0.09 | 0.16 | 0.07 | 0.13 | 0.07 | 6.64±0.81 | 6.1 | 3.5 | 3.8 | 2.8 | 260.37±8.55 | 31.92 | 40.68 | 29.01 | 26.11 |
| CTWA | 0.10±0.02 | 0.03 | 0.03 | 0.08 | 0.1 | 7.17±1.29 | 5.4 | 1.7 | 5.4 | 2.1 | 280.62±10.19 | 40.86 | 41.25 | 37.23 | 15.47 |

**Table S4.** Total N and PO_4_^3-^ content of raw and nanocomposite treated effluents

| Effluent | Total N (mg/L) | MagNPs | BioMag | MagNPs-CMC | BioMag-CMC | PO_4_^3-^ (mg/L) | MagNPs | BioMag | MagNPs-CMC | BioMag-CMC |
| --- | --- | --- | --- | --- | --- | --- | --- | --- | --- | --- |
| PCUWA | 82.48±5.17 | 72 | 71.2 | 33.7 | 69.5 | 0.77±0.05 | 0.32 | 0.21 | 0.38 | 0.33 |
| TUWK | 9.57±1.20 | 5.1 | 9.1 | 10.7 | 6 | 0.05±0.01 | 0.1 | 0.08 | 0.12 | 0.11 |
| SUWK | 26.45±2.53 | 21.4 | 24.4 | 11.4 | 30.3 | 0.43±0.09 | 0.28 | 0.21 | 0.19 | 0.16 |
| MUWA | 23.10±3.14 | 8.1 | 20.9 | 14.3 | 24.2 | 1.87±0.23 | 0.46 | 0.21 | 1.78 | 0.27 |
| PTWK | 7.21±2.33 | 3.7 | 8.9 | 7 | 10.1 | 0.53±0.08 | 0.41 | 0.21 | 0.47 | 0.28 |
| TUKW | 15.82±3.11 | 10.5 | 15.7 | 13.8 | 15.2 | 0.98±0.11 | 0.22 | 0.11 | 0.34 | 0.45 |
| BTWR | 11.37±1.64 | 9.4 | 9.7 | 13.1 | 8.8 | 1.87±0.34 | 0.91 | 0.55 | 0.69 | 0.59 |
| PUWA | 94.81±4.70 | 65.1 | 81.6 | 79.8 | 110.4 | 0.50±0.06 | 0.62 | 0.59 | 0.33 | 0.05 |
| PTK | 17.72±1.46 | 10.2 | 19.4 | 18.3 | 5.2 | 1.21±0.06 | 0.44 | 0.22 | 0.19 | 0.32 |
| FTWR | 28.33±2.19 | 13.8 | 24.5 | 19 | 26.9 | 0.93±0.21 | 0.78 | 0.18 | 0.2 | 0.27 |
| FCTWE | 11.49±2.31 | 13.8 | 15.8 | 14.9 | 17 | 0.14±0.03 | 0.05 | 0.07 | 0.04 | 0.08 |
| CMTWM | 4.07±0.85 | 2.8 | 5.7 | 5.3 | 4 | 0.19±0.01 | 0.05 | 0.02 | 0.04 | 0.02 |
| CTWA | 34.23±4.21 | 30.1 | 32.8 | 30.2 | 35.9 | 0.04±0.00 | 0.01 | 0.01 | 0.03 | 0 |

**Table S5.** Capacity of the nanoparticles to sequester metal ions

| **Wastewaters** | Uptake capacity of adsorbent (mg/g) | | | | | | | | | | | |
| --- | --- | --- | --- | --- | --- | --- | --- | --- | --- | --- | --- | --- |
|  | BioMag-CMC | | | MagNPs-CMC | | | BioMag | | | MagNPs | | |
|  | Cd | Cr | Pb | Cd | Cr | Pb | Cd | Cr | Pb | Cd | Cr | Pb |
| PCUWA | 0.49 | 0.05 | 0.008 | 0.4 | 0.05 | 0.001 | 0.43 | 0.07 | 0.005 | 0.46 | 0.03 | 0.011 |
| TUWK | 0.09 | 0.04 | 0.007 | 0.07 | 0.08 | 0.009 | 0.19 | 0.04 | 0.01 | 0.06 | 0.02 | 0.013 |
| SUWK | 0.02 | 0.08 | 0.014 | 0.05 | 0.07 | 0.011 | 0.06 | 0.09 | 0.006 | 0.04 | 0.07 | 0.006 |
| MUWA | 0.01 | 0.06 | 0.011 | 0.01 | 0.11 | 0.009 | 0.02 | 0.02 | 0.014 | 0.03 | 0.08 | 0.018 |
| PTWK | 0.03 | 0.1 | 0.009 | 0.06 | 0.08 | 0.011 | 0.04 | 0.14 | 0.016 | 0.05 | 0.1 | 0.008 |
| TUKW | 0.04 | 0.12 | 0.06 | 0.06 | 0.09 | 0.016 | 0.07 | 0.12 | 0.039 | 0.05 | 0.18 | 0.022 |
| BTWR | 0.18 | 0.06 | 0.005 | 0.2 | 0.07 | 0.008 | 0.23 | 0.11 | 0.011 | 0.23 | 0.1 | 0.008 |
| PUWA | 0.47 | 0.01 | 0.01 | 0.52 | 0.09 | 0.012 | 0.41 | 0.11 | 0.011 | 0.46 | 0.04 | 0.016 |
| PTK | 0.01 | 0.09 | 0.023 | 0.01 | 0.03 | 0.015 | 0.03 | 0.06 | 0.024 | 0.04 | 0.07 | 0.022 |
| FTWR | 0.17 | 0.09 | 0.01 | 0.18 | 0.04 | 0.015 | 0.18 | 0.02 | 0.009 | 0.12 | 0.02 | 0.012 |
| FCTWE | 0.24 | 0.05 | 0.007 | 0.31 | 0.09 | 0.008 | 0.26 | 0.02 | 0.01 | 0.28 | 0.07 | 0.005 |
| CMTWM | 0.3 | 0.07 | 0.006 | 0.24 | 0.09 | 0.01 | 0.27 | 0.07 | 0.009 | 0.29 | 0.04 | 0.007 |
| CTWA | 0.47 | 0.05 | 0.012 | 0.49 | 0.08 | 0.004 | 0.48 | 0.08 | 0.014 | 0.51 | 0.04 | 0.005 |
| Average uptake capacity | 0.48±  0.014 | 0.05±  0.00 | 0.01±  0.0028 | 0.45±  0.064 | 0.065±  0.0212 | 0.0025±  0.0021 | 0.455±  0.0354 | 0.075±  0.0071 | 0.0095±  0.0064 | 0.485±  0.0354 | 0.035±  0.007 | 0.008±  0.0042 |
|  | 0.181±0.015 | | | 0.173±0.029 | | | 0.180±0.016 | | | 0.176±0.016 | | |

**Table S6.** Capacity of the nanoparticles to sequester pesticide residues

| SN | Pesticide residues | Initial concentrations  (ng/L) | Uptake capacity of adsorbent (mg/g) | | | |
| --- | --- | --- | --- | --- | --- | --- |
|  |  |  | Biomag | MagNPs | Biomag-CMC | MagNPs-CMC |
| 1 | α-BHC | 1.10 | 10.5 | 11 | 11 | 5.8 |
| 2 | β-BHC | 0.98 | 3.9 | 9.8 | 9.3 | 9.8 |
| 3 | γ-BHC | 1.01 | 3.6 | 10.1 | 9.2 | 8.4 |
| 4 | Heptachlor | 0.97 | 1.6 | -0.4 | 6.8 | 6.7 |
| 5 | δ-BHC | 0.93 | 6.3 | 7.6 | 2.3 | 7.7 |
| 6 | Aldrin | 1.03 | 4.9 | 5.6 | 8.9 | 6.5 |
| 7 | Heptachlor Epoxide | 0.99 | 9.9 | 6.6 | 9.9 | 9.9 |
| 8 | γ-chlordane | 1.07 | 8.7 | 10.7 | 2.2 | 4.1 |
| 9 | α-chlordane | 0.98 | 9.8 | 3.1 | 0.3 | 9.8 |
| 10 | Endosulfan I | 1.28 | 10.8 | 10.7 | 12.8 | 12.8 |
| 11 | P, p'-DDE | 1.16 | 9.9 | 8.1 | 0.5 | 11.2 |
| 12 | Dieldrin | 1.76 | 13.6 | 16.8 | 10.6 | 16.2 |
| 13 | Endrin | 0.89 | 8.9 | -4.1 | 2.8 | 5.9 |
| 14 | P,P'-DDD | 0.98 | 8 | 9.8 | 8.1 | 9.8 |
| 15 | Endosulfan II | 1.21 | 12.1 | 12.1 | 12.1 | 12.1 |
| 16 | P,P'-DDT | 1.37 | 13.7 | 13.7 | 11.9 | 13.7 |
| 17 | Endrin aldehyde | 1.20 | 12 | 12 | 11.2 | 12 |
| 18 | Endosulfan Sulphate | 1.11 | 11.1 | 11.1 | 2.1 | 11.1 |
| 19 | Methoxychlor | 0.90 | 9 | 9 | 9 | 9 |
| 20 | Endrin Ketone | 1.74 | 17.4 | 17.4 | 17.4 | 17.4 |
| Average uptake capacity | | | 13.95±  4.879 | 14.2±  4.525 | 14.2±  4.525 | 11.6±  8.202 |

**Table S7**. USEPA Regulatory Guidelines.

| Parameter | units | General limit |
| --- | --- | --- |
| T | °C | Below 35 |
| pH | - | 6.5–8.5 |
| EC | (µS/cm) | 2500 |
| TDS | (mg/L) | 500 |
| DO | (mg/L) | 6.0–9.5 |
| SO_4_^2-^ | (mg/L) | 250 |
| COD | (mg/L) | Below 1000 |
| BOD | (mg/L) | Below 500 |
| Cl^-^ | (mg/L) | 250 |
| PO_4_^3-^ | (mg/L) | 4 |

United State Environmental Protection Agency; Drinking Water Regulations and Health Advisories, EPA 822-R-94–001, May 1994
